# Postoperative Pain after Laparoscopic Cholecystectomy in a Tertiary Care Center: A Descriptive Cross-sectional Study

**DOI:** 10.31729/jnma.8719

**Published:** 2024-08-31

**Authors:** Binod Bade Shrestha, Gajal Lakhe, Pradeep Ghimire

**Affiliations:** 1 Department of General Surgery, Manipal College of Medical Sciences, Phulbari, Pokhara, Nepal; 2 Department of Anesthesia, Manipal College of Medical Sciences, Phulbari, Pokhara, Nepal

**Keywords:** *cholecystectomy*, *laparoscopic*, *pain*, *post-operative*

## Abstract

**Introduction:**

Laparoscopic cholecystectomy, being minimally invasive, is widely accepted in comparison to open cholecystectomy. The major benefits are small incision, less wound pain, rapid recovery, shorter hospital stay and earlier return to activities. Although, trauma and injury are limited in laparoscopic cholecystectomy; it is not a pain free surgery. Hence, we aimed to find out the prevalence of pain at wound site after laparoscopic cholecystectomy at various time intervals in post-operative period.

**Methods:**

The descriptive cross-sectional study was conducted among 125 American Society of Anesthesiologists grade I/II patients, with diagnosis of symptomatic gallstone disease from October 2022 to September 2023 in a tertiary care hospital after ethical approval was obtained from Institutional Research Committee (Reference number: MEMG/483/IRC). Total sampling was done in this study. The post-operative pain at wound site was measured at 12, 24, 36 and 48 hours. Data were analyzed using SPSS 21.0.

**Results:**

At 12 hours postoperatively, 2 (1.60%) patients complained of severe pain, 120 (96%) patients reported moderate pain and 3 (2.40%) patients expressed their pain as being mild. Likewise, at 24, 36 and 48 hours postoperatively, none of the patients suffered from severe pain. At 24 hours post-operative, 105 (84%) patients reported moderate pain which gradually declined over 48 hours. At 36 and 48 hours post-operative mild pain was reported by 85 (68%) and 117 (93.60%) patients respectively. The moderate pain was complained by 40 (32%) and 8 (6.40%) patients respectively.

**Conclusions:**

The majority of patients suffered from mild to moderate pain after laparoscopic cholecystectomy, the intensity of which decreased over 48 hours.

## INTRODUCTION

Laparoscopic cholecystectomy (LC) is considered a gold standard procedure for management of cholelithiasis. The major benefits being shorter hospital stays, early recovery and less postoperative pain.^1^ Laparoscopic cholecystectomy being a minimally invasive surgery leads to less tissue trauma. However, it is not a pain free technique. Hence, postoperative pain remains a significant concern for many patients undergoing laparoscopic cholecystectomy. It is an important factor that leads to prolonged admissions or readmissions.^2^ Immediately after surgery patients might experience some pain and/or discomfort, the duration and severity of which might vary from patient to patient.

Pain after laparoscopic cholecystectomy could be due to tissue injury in the anterior abdominal wall during insertion of trocar, visceral pain and shoulder tip pain due to diaphragmatic irritation caused by spillage of blood, bile or peritoneal stretching during pneumoperitoneum.^3^

Despite postoperative pain being an important issue after laparoscopic cholecystectomy,^2^ we could not find studies done to assess it in our settings. Meanwhile, laparoscopic cholecystectomy is one of the most commonly conducted surgeries in our institution.

Thus, the aim of this study was to assess the severity and characteristics of postoperative pain in patients undergoing laparoscopic cholecystectomy.

## METHODS

This descriptive cross-sectional study was conducted in the Department of General Surgery, Manipal College of Medical Sciences, Pokhara, Nepal from October 20, 2022 to September 19, 2023. The study was conducted after the approval from Institutional Review Board (Reference number: MEMG/483/IRC). Prospective data collection was done for which the written and informed consent was obtained from all cases. The patients aged 18 to 65 years, who belonged to American Society of Anesthesiologists (ASA) grade I and II,^4^ scheduled for laparoscopic cholecystectomy for symptomatic gallstone disease under general anesthesia were enrolled in the study. Patients with acute pancreatitis, acute cholecystitis, pregnancy, history of peritonitis and the cases in whom surgery was converted to open cholecystectomy or in whom common bile duct was explored were excluded. A total of 125 laparoscopic cholecystectomy was conducted during the study period and all of those cases were enrolled in our study.

The pre-anesthetic evaluation was done a day prior to surgery during which patients were educated about Visual Analogue Scale (VAS) which ranged from 0-10, where 0 meant no pain and 10 referred to the worst pain one has ever experienced. They had to grade their postoperative pain as per the VAS scoring system at 12, 24, 36 and 48 hours. The pain was categorized as mild (0-3), moderate (4-7) and severe (8-10) based on VAS.^5^

The general anesthesia was standardized for all cases. In the operation theater, an intravenous line was secured. Injection fentanyl 2 pg/kg was given for analgesia. Induction of anesthesia was done with propofol 2-2.5 mg/kg and muscle paralysis for laryngoscopy and intubation was obtained using succinylcholine 2 mg/kg. The female and male patients were intubated with size 7 mm and 8 mm endotracheal tubes respectively. The port site was infiltrated with 10 ml 0.25% plain bupivacaine at the beginning of surgery. Surgery was performed by a single surgeon using standard four working ports technique. Anesthesia was maintained by using O2 along with Isoflurane and additional doses of vecuronium when needed. End tidal CO2 was monitored and maintained between 35-40 mm of Hg. During laparoscopy, intra abdominal pressure was maintained between 10-12 mm of Hg. At the end of surgery, CO2 was carefully evacuated by manual compression of abdomen with open trocars and injection ketorolac 30 mg plus ondansetron 4 mg were given. The residual neuromuscular blockade was reversed with neostigmine 0.05 mg/kg and glycopyrrolate 0.01 mg/kg prior to extubation.

Injection paracetamol 1 gm 8 hourly, ketorolac 30 mg 8 hourly and tramadol 50 mg IV if needed were prescribed for postoperative analgesia. We recorded postoperative pain at 12, 24, 36 and 48 hours. The postoperative VAS score was evaluated by surgery residents not involved in study. The duration of surgery, intraoperative bile spillage, calculi spillage and pain at shoulder tip were noted.

Data was analyzed using SPSS version 21.0. Descriptive statistics were performed and results expressed as mean±SD, frequency and percentage wherever applicable.

## RESULTS

A total of 125 patients with postoperative pain after laparoscopic cholecystectomy were enrolled in study. The mean age of patients was 44.81±12.05 years. The study population included 37 (29.60%) males and 88 (70.40%) females.

Most of the patients complained of mild to moderate pain up-to 48 hours in postoperative period. At twelve hours postoperatively, 2 (1.6%) patients complained of severe pain whereas 120 (96%) patients reported moderate pain and 3 (2.4%) patients expressed their pain as being mild. Likewise, at 24, 36 and 48 hours postoperatively; none of the patients suffered from severe pain. At 24 hours postoperative 105 (84%) patients reported moderate pain which gradually declined over 48 hours (Table 1).

Among 125 patients, 2 (1.60%) cases complained of pain at the tip of shoulder and 123 (98.40%) didn't experience pain at shoulder tip (Table 2).

**Table 1:** Postoperative pain at various time interval (n = 125).

Time interval	Severity	VAS^*^ n(%)
12 hour	Mild	3 (2.40)
	Moderate	120 (96)
	Severe	2 (1.60)
24 hour	Mild	20 (16)
	Moderate	105 (84)
	Severe	-
36 hour	Mild	85 (68)
	Moderate	40 (32)
	Severe	-
48 hour	Mild	117 (93.60)
	Moderate	8 (6.40)
	Severe	-

* VAS: Visual analogue scale

**Table 2:** Various surgical parameters (n = 125).

Duration of surgery (min)	30.94±7.69
Shoulder tip pain	n (%)
Yes	2 (1.60)
No	123 (98.40)
**Bile spillage**	
Yes	9 (7.20)
No	116 (92.80)
**Calculi spillage**	
Yes	1 (0.80)
No	124 (99.20)
**Gall bladder extraction site**	
Umbilical	78 (62.40)
Epigastric	47 (37.60)
**Need of opioid**	
Yes	2 (1.60)
No	123 (98.40)

## DISCUSSION

The major benefit of laparoscopic cholecystectomy is decreased postoperative pain as there is minimal tissue damage as compared to open cholecystectomy. However, it is not a pain free surgery. Our study found that pain was mostly mild to moderate in severity in our patients up to 48 hours postoperative period with only two patients complaining of severe pain in the first twelve hours postoperative. The port site infiltration with local anesthetic might also have reduced the somatic component of pain as we infiltrated 0.25% plain Bupivacaine at the port site prior to incision in all our cases. Likewise, the postoperative consumption of opioid was minimal in our study.

The past studies have documented 60% to 76.7% of patients complained of moderate or severe pain during 24 postoperative hours.^6,7^ In the present study, we also found that moderate pain dominated over mild and severe pain.

We had infiltrated port site with 0.25% bupivacaine in all our patients. This might be the reason for the lower prevalence of severe postoperative pain in our study. Only 2 (1.63%) patients suffered from severe pain at twelve hours post-surgery requiring added opioid analgesia. While using high doses of opioid intraoperative pain may increase because of rapid elimination and development of acute tolerance.^8^ Likewise, past investigators have found that pain after laparoscopic cholecystectomy in patients whose wound were infiltrated with local anesthetic reported pain as moderate in nature and the consumption of rescue analgesics in the form of opioid was less.^9^

Previous study have reported severe pain in 44.9% of patients post-laparoscopic cholecystectomy which is higher than our findings.^10^ This discrepancy is probably due to skin infiltration with local anesthetic in our cases.

Patients frequently complain of pain in the shoulder region, back and at incision site. The past study have shown that incisional pain dominated in incidence and intensity compared with visceral pain, which in turn dominated over shoulder pain.^7^ In our study, even though we didn't study the prevalence of visceral pain, pain at incision site dominated over the pain at shoulder tip. Postoperative shoulder pain is often complained by patient after laparoscopic cholecystectomy which is believed to be due to phrenic nerve neuropraxia due to stretching of diaphragm following carboperitoneum, which causes referred pain.^11^ However, there are few theories stating shoulder pain is secondary to excessive traction of the triangular ligament and over-stretching of the diaphragmatic fibers due to insufflation. One of the past study have documented that shoulder and sub-diaphragmatic pain occurs in about 12% to 60% of patients.^12,13^ Our findings are in contrast to former study. We noted that pain at the shoulder tip was present only in 1.6% of patients. The reason is probably because CO_2_ was carefully evacuated at the end of surgery and all cases were done by same surgeon which might have reduced inter-individual variability in the procedure. There are several prior studies which support that evacuating residual CO_2_ reduces the frequency and intensity of post laparoscopic shoulder pain in the first 24 hours after laparoscopic surgery.^14-16^ The other probable reason could be due to minimal biliary leakage and calculi spillage in our study as presented in Table 1. Intraoperative bile spillage following iatrogenic gallbladder perforation is common in early learning curve. Morbidity may include surgical site infection, ileus of intestine, intraperitoneal abscess or no symptoms at all.^17^ Studies have shown that postoperative pain and shoulder tip pain have direct relation to the spillage of bile.^18^

Former studies have highlighted that pain intensifies the most during the first few postoperative hours and usually declines after two or three days.^7,19^ The findings of our study are alike past studies as it is clearly evident from Table 2 that pain intensity gradually declined over 48 hours. One of the strengths of our study is that all cases were performed by a single surgeon because the experience and skill of surgeon affects tissue handling and closure techniques which has direct impact likelihood of postoperative pain.

Although laparoscopic cholecystectomy is minimally invasive several studies have demonstrated that patient complains of pain of varying intensity despite the use of various modalities of pain management, thus emphasizing the importance and necessity of adequate pain control in the initial postoperative period because poorly managed acute postoperative pain often leads to persistent chronic pain.^3^-^10^'^20^-^21^ This highlights the importance of multimodal analgesic technique that needs to be incorporated in our practice to improve the perioperative pain management which will enhance the patient satisfaction, decrease hospital stay, early recovery, discharge and earlier return to activities of daily living. In past study of evidence-based pain management after laparoscopic cholecystectomy even providing analgesic prior days to surgery has shown to decrease postoperative pain.^1^ With respect to recent advance in pain management, multimodal analgesia has been used in controlling and decreasing postoperative pain facilitating early recovery and ambulation. Infiltration of local anesthesia at port site solely or even added with infiltration at liver bed at the end of surgery and transversus abdominis plane block has shown to decrease prevalence of acute postoperative pain following laparoscopic cholecystectomy.^8,22,23^

There are certain limitations of this study. We have failed to assess visceral pain, severity of shoulder tip pain, dose of rescue analgesic, specify which wound site was most painful and postoperative nausea-vomiting. We suggest a further study needs to be conducted incorporating these aspects in our patients undergoing laparoscopic cholecystectomy.

## CONCLUSIONS

Our study demonstrates that laparoscopic cholecystectomy, while not entirely pain-free, majority of the patients suffered from mild to moderate pain after laparoscopic cholecystectomy, the intensity of which decreased over 48 hours and there was minimal requirement of opioid, with severe pain being rare.
